# Safety and Treatment Discontinuation of Novel Hormonal Therapies in Metastatic Hormone-Sensitive Prostate Cancer: An Exploratory Real-World Study

**DOI:** 10.3390/cancers18132144

**Published:** 2026-07-03

**Authors:** Irene Millan-Ramos, Alberto Zambudio-Munuera, Miguel Herraez-Marcos, Antonio Jimenez-Pacheco, Francisco Gutierrez-Tejero, Miguel Arrabal-Martin, Miguel Angel Arrabal-Polo

**Affiliations:** 1 Urology Department, San Cecilio University Hospital, 18016 Granada, Spain; 2 Instituto Investigación Biosanitaria de Granada (IBS), 18012 Granada, Spain

**Keywords:** metastatic hormone-sensitive prostate cancer, mHSPC, novel hormonal therapies, real-world evidence, adverse events, treatment discontinuation, polypharmacy, drug–drug interactions

## Abstract

**Background/Objectives:** Treatment intensification with novel hormonal therapies is now standard in metastatic hormone-sensitive prostate cancer (mHSPC), but real-world patients are often more heterogeneous than those included in pivotal trials. This study aimed to describe clinical characteristics, safety, treatment discontinuation, disease progression, polypharmacy, and clinically documented drug–drug interactions in a real-world mHSPC cohort. **Methods:** We conducted a retrospective observational study including 109 patients with mHSPC who initiated abiraterone, enzalutamide, apalutamide, or docetaxel-based triplet regimens between January 2015 and November 2025. Outcomes included adverse events, discontinuation, PSA50 response at 3 months, time to progression and overall survival. Descriptive analyses and Kaplan–Meier estimates were performed. **Results:** Apalutamide was the most frequent first-line treatment (56.9%), followed by abiraterone (23.9%), enzalutamide (11.9%), darolutamide-based triplet therapy (3.7%), and abiraterone-based triplet therapy (3.7%). Median age was 73 years, and median baseline PSA was 16.1 ng/mL. De novo metastatic disease was present in 69.7% of patients, ISUP grade 4–5 disease in 58.7%, high-risk disease according to LATITUDE criteria in 45.9%, and high-volume disease according to CHAARTED criteria in 38.5%. The median Charlson Comorbidity Index was 4, and polypharmacy was observed in 68.8%. Adverse events occurred in 56.0%, and non-death treatment discontinuation occurred in 22.0%. No documented drug–drug interactions requiring treatment modification were recorded. PSA50 response was achieved by 97.2%. Thirteen patients (11.9%) progressed and 18 (16.5%) died. Median time to progression was not reached, and median overall survival was 53.2 months. **Conclusions:** Novel hormonal therapies were used in a clinically heterogeneous real-world mHSPC cohort. The findings support individualized treatment assessment and should be interpreted as descriptive and exploratory.

## Simple Summary

Treatment for metastatic hormone-sensitive prostate cancer has changed substantially in recent years with the introduction of novel hormonal therapies administered with androgen deprivation therapy, with or without docetaxel. However, patients treated in routine clinical practice are often more complex than those included in clinical trials. In this retrospective real-world study, we analyzed 109 patients treated with abiraterone, enzalutamide, apalutamide, or docetaxel-based triplet regimens. We focused on adverse events, treatment discontinuation, disease progression, polypharmacy, and potential drug–drug interactions. Our findings suggest that these treatments are feasible in daily clinical practice, although toxicity and discontinuation patterns differed between treatment strategies. These results highlight the importance of individualized treatment selection, particularly in patients with comorbidities, polypharmacy, or an increased risk of toxicity.

## 1. Introduction

Metastatic hormone-sensitive prostate cancer (mHSPC) has undergone a major paradigm shift over the past decade [1,2]. Significant improvements in overall survival and progression-free survival have been achieved with the incorporation of novel hormonal therapies in addition to androgen deprivation therapy (ADT), which had previously constituted the standard treatment as monotherapy [2,3,4]. Current treatment strategies include these hormonal therapies and, in selected patients, chemotherapy with docetaxel [1,5,6,7,8].

Doublet combinations of abiraterone, enzalutamide, and apalutamide with ADT have become established as part of the standard of care [2,3,4,7,8]. Likewise, triplet regimens combining docetaxel with darolutamide or abiraterone have become part of the standard of care in selected patients [5,6]. Pivotal trials have demonstrated significant clinical benefits with these therapeutic strategies [9].

However, the results of pivotal clinical trials do not always reflect what is observed in real-world clinical practice [10]. Patients treated in routine clinical practice are more heterogeneous and more often have comorbidities and polypharmacy, factors that may influence treatment tolerability and continuation [11]. In this context, real-world data are particularly relevant for describing the safety profile, associated adverse events, treatment discontinuation, and patterns of disease progression observed in routine clinical practice [10,12].

In this setting, the aim of this exploratory study was to describe the clinical and oncological characteristics of a cohort of patients with metastatic hormone-sensitive prostate cancer (mHSPC) treated with novel hormonal therapies, either in combination with docetaxel or without it, and to assess efficacy and safety outcomes in real-world clinical practice. Specifically, disease progression, adverse events, treatment discontinuation, and potential drug–drug interactions were evaluated.

## 2. Methods

A retrospective observational study was conducted in patients with metastatic hormone-sensitive prostate cancer (mHSPC) who initiated treatment with novel hormonal therapies in combination with ADT, either with or without docetaxel, between January 2015 and November 2025. A total of 109 patients were included. The reporting of this observational study was informed by the STROBE recommendations and, given the use of routinely collected clinical data, by the RECORD statement [13,14].

Patients with a diagnosis of mHSPC who initiated one of the following treatment regimens were eligible for the study: abiraterone, enzalutamide, or apalutamide in combination with ADT without docetaxel, as well as abiraterone or darolutamide in combination with ADT and docetaxel. All patients had sufficient clinical information for analysis.

Baseline demographic and oncological variables, as well as efficacy and safety outcomes, were collected. The variables analyzed included age, baseline PSA levels and PSA changes during treatment, metastatic volume according to CHAARTED criteria, metastatic risk according to LATITUDE criteria, number of metastases (defined as the total number of metastatic lesions identified in baseline staging imaging), site of metastasis, type of metastatic presentation (synchronous or metachronous), Charlson Comorbidity Index, polypharmacy, excessive polypharmacy, and concomitant medication categories, including cardiovascular drugs, statins, antidiabetic drugs, psychotropic drugs, antiplatelet therapy and anticoagulant therapy. Polypharmacy was defined as the concomitant use of 5 or more medications, and excessive polypharmacy as the use of 10 or more medications. The Charlson Comorbidity Index was calculated excluding metastatic prostate cancer as the index disease. Concomitant medication categories were recorded at treatment initiation.

The main efficacy outcome was disease progression, classified as biochemical, radiological or clinical progression according to a predefined operational definition applied during retrospective chart review. Biochemical progression was defined as a PSA increase of at least 25% and at least 2 ng/mL above the PSA nadir, confirmed by a subsequent PSA determination when available. Radiological progression was defined as progression of known metastatic lesions or the appearance of new metastatic lesions on follow-up imaging, according to the radiology report and routine clinical interpretation. Clinical progression was defined as symptomatic deterioration, clinical worsening attributable to prostate cancer, or the need to modify systemic treatment because of disease progression.

Additional exploratory effectiveness outcomes included PSA50 response at 3 months, time to progression and overall survival. PSA50 response at 3 months was defined as a reduction of at least 50% in PSA levels from treatment initiation to the 3-month assessment. Time to progression was defined as the time from treatment initiation to documented biochemical, radiological or clinical progression. Patients without progression were censored at the date of last follow-up. Overall survival was defined as the time from treatment initiation to death from any cause, with living patients censored at the date of last follow-up.

Safety outcomes included adverse events, temporary or permanent treatment discontinuation, and clinically documented drug–drug interactions requiring treatment modification. Adverse events were identified through review of the medical records.

Descriptive statistical analysis was performed. Continuous variables were expressed as mean and standard deviation or median and interquartile range, according to their distribution, while categorical variables were presented as absolute frequencies and percentages. Time-to-event outcomes, including time to progression and overall survival, were estimated using the Kaplan–Meier method. Given the retrospective observational design, the non-randomized allocation of treatment and the small size of several treatment subgroups, all treatment-specific analyses were considered descriptive and exploratory. No formal statistical comparisons between treatment groups, including log-rank tests or Cox regression models, were performed. The analysis was performed using Jamoviversion 2.6.45.0.

The study was approved by the corresponding Research Ethics Committee (code [RWD-CPHSM-NTH-2026]). Due to the retrospective nature of the study, the requirement for informed consent was waived.

## 3. Results

### 3.1. Patient Characteristics

A total of 109 patients were included. Most patients received apalutamide as first-line treatment (62/109; 56.9%), followed by abiraterone (26/109; 23.9%), enzalutamide (13/109; 11.9%), darolutamide-based triplet therapy (4/109; 3.7%), and abiraterone-based triplet therapy (4/109; 3.7%). The median age was 73 years (IQR 11). The median baseline PSA level was 16.1 ng/mL (IQR 48.2; range 0.2–3119 ng/mL). Polypharmacy involving the use of 5 or more concomitant medications was present in 68.8% of patients, while 36.7% were receiving 10 or more concomitant medications.

Polypharmacy and concomitant medication categories at treatment initiation are summarized in Table 1. Cardiovascular drugs were the most frequent concomitant medication category, followed by statins, while polypharmacy was frequent in the overall cohort.

The cohort showed a relevant comorbidity burden, with a median Charlson Comorbidity Index of 4 (IQR 2). The mean Charlson Comorbidity Index was 3.85 (SD 1.76), with a range from 0 to 10.

### 3.2. Oncological Characteristics

Regarding oncological characteristics, 69.7% of patients had de novo metastatic disease, whereas 30.3% had metachronous disease. ISUP grade 4–5 histology was observed in 58.7% of cases, and 45.9% of the cohort was classified as high-risk according to LATITUDE criteria. The median number of metastases was 5 (IQR 8; range 1–50). Initial staging was performed using computed tomography (CT) and bone scan in 55% of patients, PSMA PET in 29%, and choline PET in 15%.

According to CHAARTED criteria, 67 patients (61.5%) had low-volume disease and 42 patients (38.5%) had high-volume disease.

Metastatic location was classified into seven categories: nodal-only, bone-only, nodal plus visceral, visceral-only, nodal plus bone, visceral plus bone, and nodal plus visceral plus bone metastases.

When metastatic involvement was considered by site, regardless of whether it was isolated or combined with other metastatic locations, 67 patients (61.5%) had nodal involvement, 95 patients (87.2%) had bone metastases and 13 patients (11.9%) had visceral metastases. Overall, the most frequent metastatic pattern was combined nodal and bone involvement, observed in 51 patients (46.8%), followed by bone-only disease, observed in 35 patients (32.1%).

Baseline demographic and oncological characteristics are shown in Table 2. Metastatic location at treatment initiation is summarized in Table 3. Polypharmacy and concomitant medication categories at treatment initiation are shown in Table 1.

### 3.3. Safety Outcomes

Adverse events were observed in 61 patients (56.0%). By treatment group, adverse events occurred in 51.6% of patients treated with apalutamide, 42.3% of those treated with abiraterone, 92.3% of those treated with enzalutamide, 50.0% of patients receiving abiraterone-based triplet therapy, and 100% of those receiving darolutamide-based triplet therapy, although the sample size in these last two groups was small. The most frequent toxicities were asthenia, rash, and hot flashes in the apalutamide group; edema and metabolic disturbances in the abiraterone group; and predominantly asthenia in the enzalutamide group.

In the darolutamide plus docetaxel and ADT triplet group, adverse events were recorded in all patients (4/4, 100%). The most frequently recorded adverse events in this subgroup were hypertransaminasemia, asthenia, edema and neurotoxicity/neuropathy, each reported in two patients. Other adverse events included cutaneous lesions, dryness, hot flashes, constipation, febrile neutropenia, alopecia, dyspnea and acute urinary retention, each reported in one patient.

In the abiraterone plus docetaxel and ADT triplet group, adverse events were recorded in two of four patients (50%). The reported adverse events were dysgeusia in one patient and edema in one patient. No adverse events were recorded in the remaining two patients.

No clinically documented drug–drug interactions requiring treatment modification were recorded in the medical records.

Adverse events according to treatment group are summarized in Table 4.

### 3.4. Disease Progression

Disease progression was assessed according to the information recorded in the medical records, including radiological, clinical, or biochemical progression. Progression was also recorded as a reason for treatment discontinuation when applicable. In addition, PSA values decreased over time in most treatment groups, although interpretation of long-term PSA values should be cautious because of the low number of evaluable patients at later time points, particularly in the triplet therapy groups. Median PSA evolution over time is shown in Table 5. PSA50 response at 3 months was achieved by 106 of 109 patients (97.2%), whereas 3 patients (2.8%) did not achieve a PSA reduction of at least 50% at 3 months. PSA50 response by treatment group is summarized in Table 6.

Time-to-event outcomes were assessed in the overall cohort, with no missing values for the time variables. The median observed time for the overall survival analysis was 19.8 months, and the median observed time for the progression analysis was 17.1 months. During follow-up, 13 patients (11.9%) experienced disease progression and 18 patients (16.5%) died.

Median time to progression was not reached in the overall cohort. The estimated probability of remaining free from documented progression was 94.7% (95% CI 90.2–99.4) at 12 months and 81.2% (95% CI 71.4–92.4) at 36 months. Median overall survival was 53.2 months, with the upper limit of the 95% confidence interval not estimable. The estimated overall survival probability was 96.7% (95% CI 93.2–100.0) at 12 months and 73.1% (95% CI 62.4–85.5) at 36 months.

Exploratory time-to-event outcomes by treatment group are summarized in Table 7. These subgroup analyses were descriptive and exploratory, given the low number of patients and events in some treatment groups. Therefore, no formal statistical comparisons between treatment groups were performed.

### 3.5. Treatment Discontinuation

Treatment discontinuation was documented in 24 patients (22.0%): 10 in the abiraterone group, 5 in the enzalutamide group, 5 in the apalutamide group, 3 in the darolutamide-based triplet therapy group, and 1 in the abiraterone-based triplet therapy group. Reasons for treatment discontinuation according to treatment group are summarized in Table 8.

## 4. Discussion

In this exploratory real-world study, we described a cohort of patients with metastatic hormone-sensitive prostate cancer treated with novel hormonal therapies. Rather than providing comparative evidence between therapeutic strategies, our findings offer a descriptive view of treatment patterns, tolerability, treatment discontinuation and exploratory effectiveness outcomes, including PSA response and time-to-event outcomes, in a clinically heterogeneous population. This is particularly relevant in routine practice, where treatment decisions are often influenced not only by disease characteristics, but also by comorbidity, polypharmacy, clinical complexity and the anticipated risk of toxicity [15,16,17].

The baseline profile of the cohort reflects the complexity of real-world mHSPC management. A high proportion of patients had de novo metastatic disease, and a substantial percentage were classified as high-risk according to LATITUDE criteria, supporting the presence of a relevant oncological burden [18]. In addition, polypharmacy was frequent, which may influence treatment tolerability, adherence and the ability to maintain intensified systemic therapy over time [19,20,21]. These factors are often underrepresented in pivotal trials and may partly explain why real-world treatment patterns and discontinuation profiles can differ from those observed in more selected trial populations [18,22].

Regarding safety, the observed patterns should be interpreted as real-world tolerability signals rather than as formal comparisons between drugs. The observed variation in adverse event profiles across treatment groups may reflect not only drug-specific toxicity, but also baseline patient characteristics, comorbidity burden, concomitant medications, physician preferences and follow-up intensity. This interpretation is consistent with previous real-world studies describing variability in the tolerability of novel hormonal therapies in mHSPC [23,24,25]. The tolerability of novel hormonal therapies in mHSPC does not depend solely on the drug administered, but also on patients’ baseline characteristics, associated comorbidity, and the clinical management of adverse events [18,23,26]. Recent studies have suggested that a multidisciplinary approach and the early identification of toxicities may favor better treatment continuity, especially in patients receiving apalutamide [26,27].

Treatment discontinuation represents a relevant issue in real-world clinical practice, because it integrates several dimensions beyond oncological efficacy alone, including tolerability, clinical deterioration, patient vulnerability and decision-making in complex clinical scenarios. In our cohort, the reasons for discontinuation varied across treatment strategies, but these findings should be interpreted descriptively given the small size of some subgroups. Previous real-world studies have similarly suggested that treatment persistence may be influenced by toxicity, dose adjustments and baseline clinical complexity, particularly among patients receiving apalutamide or more intensified regimens [28,29,30,31]. Overall, these findings support the need for individualized treatment selection and close follow-up when implementing intensified systemic therapy in routine clinical practice.

Despite the clinical relevance of these findings, they should be interpreted with caution, as this is a descriptive and exploratory study that was not designed to establish robust comparisons between therapeutic strategies. The results should therefore be understood primarily as a description of patterns of use, safety, treatment discontinuation and exploratory effectiveness outcomes observed in a real-world cohort, rather than as conclusive evidence of differences between treatments.

This study has several limitations that should be acknowledged. First, it was a retrospective, descriptive, and exploratory study, with the inherent limitations related to selection bias and reliance on information recorded in the medical records. Second, the sample size was limited in some therapeutic subgroups, particularly in the triplet regimens, which restricts the robustness of comparisons across strategies. Therefore, treatment-specific results should be interpreted as descriptive and exploratory, and no causal or definitive comparative conclusions can be drawn.

In addition, adverse events were retrospectively identified through review of routine medical records and were not prospectively graded according to standardized CTCAE criteria. As a result, the frequency and severity of adverse events may have been influenced by the completeness of clinical documentation, and low-grade or transient toxicities may have been underreported. Therefore, the safety findings should be interpreted as descriptive real-world data and should not be directly compared with toxicity rates from prospective clinical trials. Similarly, potential drug–drug interactions were not systematically assessed using a standardized interaction-checking tool. Only clinically documented drug–drug interactions requiring treatment modification were recorded from the medical records. Therefore, the frequency of potential drug–drug interactions may have been underestimated, particularly given the high prevalence of polypharmacy and excessive polypharmacy in the cohort.

The long study period should also be considered when interpreting the results. Between 2015 and 2025, the therapeutic landscape of mHSPC changed substantially, and the availability of intensified systemic therapies in Spain evolved over time. Spanish therapeutic positioning reports for mHSPC were published for abiraterone in 2020, apalutamide in 2021, enzalutamide in 2022 and darolutamide in combination with docetaxel and ADT in 2024 [32,33,34,35]. As a result, treatment selection, baseline staging, patient characteristics and follow-up patterns may have been influenced by the calendar period in which patients were treated. This temporal heterogeneity may also partly explain the imbalance in treatment subgroup sizes and further supports the descriptive and exploratory interpretation of treatment-specific findings.

Furthermore, although exploratory Kaplan–Meier analyses for time to progression and overall survival were performed, the low number of observed events and the small size of several treatment subgroups limit the precision of these estimates, particularly for subgroup-level interpretation. Progression assessment was based on retrospective review of routine clinical practice and medical record documentation. Although an operational definition of biochemical, radiological and clinical progression was applied for the purposes of this study, formal standardized progression criteria were not prospectively applied across all patients. This may have introduced heterogeneity in the timing, detection and classification of progression events.

Another relevant limitation is the heterogeneity of baseline staging imaging. During the study period, baseline staging was performed according to routine clinical practice using different imaging modalities, including CT/bone scan, choline PET and PSMA PET. These modalities have different diagnostic sensitivity, which may have influenced baseline disease classification, including metastatic volume and metastatic location, as well as the timing or detection of disease progression. Therefore, this heterogeneity should be considered when interpreting the descriptive disease characteristics and exploratory effectiveness outcomes.

Finally, the single-center nature of the study may limit the generalizability of the findings to other clinical settings.

Overall, our results suggest that novel hormonal therapies administered with ADT, either with or without docetaxel, represent feasible real-world treatment options for patients with mHSPC, although tolerability and treatment discontinuation profiles may differ according to the therapeutic strategy used and patients’ baseline characteristics. In this context, individualized assessment of comorbidity, polypharmacy, and toxicity risk is particularly relevant, as these factors may influence therapeutic decision-making. Further multicenter studies with larger sample sizes and prospective study designs are needed to better define the impact of these strategies in routine clinical practice.

## 5. Conclusions

In this real-world cohort, novel hormonal therapies administered with ADT, either with or without docetaxel, were used in routine clinical practice for patients with metastatic hormone-sensitive prostate cancer and showed variable patterns of adverse events and treatment discontinuation across therapeutic strategies. Given the descriptive and exploratory nature of the study, these findings should not be interpreted as evidence of comparative differences between treatments.

Our results support the relevance of individualized treatment assessment in routine clinical practice, particularly considering patients’ baseline characteristics, comorbidity burden, polypharmacy and toxicity risk when selecting and maintaining intensified treatment strategies. Given the retrospective, single-center and exploratory nature of the study, these findings should be interpreted with caution. Prospective, multicenter studies with larger sample sizes are needed to confirm these observations and to define more precisely the impact of these strategies in real-world clinical practice.

## Figures and Tables

**Table 1:** Polypharmacy and concomitant medication categories at treatment initiation.

Variable	*n* (%)
Polypharmacy ≥ 5 medications, *n* (%)	75 (68.8)
Excessive polypharmacy ≥10 medications, *n* (%)	40 (36.7)
Cardiovascular drugs	68 (62.4)
Statins	60 (55.0)
Psychotropic drugs	35 (32.1)
Antidiabetic drugs	35 (32.1)
Antiplatelet therapy	18 (16.5)
Anticoagulant therapy	15 (13.8)

Note: Concomitant medication categories were recorded at treatment initiation and are not mutually exclusive. Polypharmacy was defined as the concomitant use of 5 or more medications, and excessive polypharmacy as the use of 10 or more medications. Percentages were calculated over the overall cohort (*N* = 109).

**Table 2:** Baseline clinical and oncological characteristics of the cohort.

Variable	Total Cohort, *n* = 109
Age, median (IQR), years	73 (11)
Baseline PSA, median (IQR), ng/mL	16.1 (48.2)
Baseline PSA, range, ng/mL	0.2–3119
De novo metastatic disease, *n* (%)	76 (69.7)
Metachronous metastatic disease, *n* (%)	33 (30.3)
ISUP grade 4–5, *n* (%)	64 (58.7)
High-risk disease according to LATITUDE criteria, *n* (%)	50 (45.9)
High-volume disease according to CHAARTED criteria, *n* (%)	42 (38.5)
Number of metastases, median (IQR)	5 (8)
Number of metastases, range	1–50
Charlson Comorbidity Index, median (IQR)	4 (2)
Charlson Comorbidity Index, mean ± SD	3.85 ± 1.76

Note: Data are presented as *n* (%) unless otherwise specified. Abbreviations: IQR, interquartile range; PSA, prostate-specific antigen; ISUP, International Society of Urological Pathology.

**Table 3:** Metastatic location at treatment initiation.

Metastatic Location	*n* (%)
Nodal-only metastases	10 (9.2)
Bone-only metastases	35 (32.1)
Nodal + visceral metastases	2 (1.8)
Visceral-only metastases	2 (1.8)
Nodal + bone metastases	51 (46.8)
Visceral + bone metastases	5 (4.6)
Nodal + visceral + bone metastases	4 (3.7)

Note: Metastatic location was categorized according to the combination of metastatic sites present at treatment initiation. Categories are mutually exclusive, so each patient was assigned to only one metastatic location category. Percentages were calculated over the overall cohort (*N* = 109).

**Table 4:** Adverse events according to treatment group.

Treatment Group	Total Patients, *n*	Patients with Adverse Events, *n* (%)	Most Frequent Adverse Events
Apalutamide	62	32 (51.6)	Asthenia, rash, hot flashes
Abiraterone	26	11 (42.3)	Edema, metabolic disturbances
Enzalutamide	13	12 (92.3)	Asthenia
Darolutamide-based triplet therapy	4	4 (100.0)	Hypertransaminasemia, asthenia, edema and neurotoxicity
Abiraterone-based triplet therapy	4	2 (50.0)	Dysgeusia and edema (1/4 each)
Total	109	61 (56.0)	—

Note: Percentages were calculated within each treatment group.

**Table 5:** Median PSA evolution over time.

Treatment	Baseline	1 Month	3 Months	6 Months	12 Months	24 Months	36 Months
Enzalutamide (*n* = 13)	33.0	0.270	0.100	0.020	0.020	0.020	0.015
Darolutamide-based triplet therapy (*n* = 4)	15.7	28.5	1.30	0.200	0.200	—	—
Abiraterone (*n* = 26)	40.3	4.00	0.710	0.335	0.170	0.620	1.96
Abiraterone-based triplet therapy (*n* = 4)	95.1	4.70	0.540	0.190	0.575	0.900	569 *
Apalutamide (*n* = 62)	8.91	0.225	0.030	0.030	0.020	0.030	0.030

Note: PSA values are expressed as median values in ng/mL. * At 36 months in the abiraterone-based triplet therapy group, the number of evaluable patients was very low; therefore, this value should be interpreted with caution. Abbreviation: PSA, prostate-specific antigen.

**Table 6:** PSA50 response at 3 months by treatment group.

Treatment Group	PSA50 Response at 3 Months, *n*/*N* (%)
Overall cohort	106/109 (97.2)
Apalutamide	62/62 (100.0)
Enzalutamide	13/13 (100.0)
Abiraterone	24/26 (92.3)
Darolutamide + docetaxel + ADT	3/4 (75.0)
Abiraterone + docetaxel + ADT	4/4 (100.0)

Note: PSA50 response was defined as a reduction of at least 50% in PSA levels from treatment initiation to the 3-month assessment.

**Table 7:** Exploratory time-to-event outcomes by treatment group.

Treatment Group	*n*	Progression Events, *n* (%)	Median Time to Progression, Months	Deaths, *n* (%)	Median Overall Survival, Months
Overall cohort	109	13 (11.9)	Not reached	18 (16.5)	53.2
Apalutamide	62	4 (6.5)	Not reached	6 (9.7)	53.2
Enzalutamide	13	0 (0.0)	Not reached	2 (15.4)	Not reached
Abiraterone	26	8 (30.8)	31.3	9 (34.6)	26.1
Darolutamide + docetaxel + ADT	4	0 (0.0)	Not reached	0 (0.0)	Not reached
Abiraterone + docetaxel + ADT	4	1 (25.0)	Not reached	1 (25.0)	Not reached

Note: Time-to-event outcomes by treatment group are descriptive and exploratory. No formal statistical comparisons between treatment groups were performed. ADT: androgen deprivation therapy.

**Table 8:** Reasons for treatment discontinuation according to treatment group.

Treatment	Progression, %	Toxicity, %	Clinical Deterioration, %	Loss to Follow-Up, %	No. of Discontinuations
Enzalutamide	0	100	0	0	5
Abiraterone	80	10	0	10	10
Apalutamide	80	0	20	0	5
Darolutamide-based triplet therapy	0	33	67	0	3
Abiraterone-based triplet therapy	100	0	0	0	1

Note: Percentages were calculated among patients with treatment discontinuation within each treatment group. Abbreviations: No., number.

## Data Availability

The data supporting the findings of this study are not publicly available due to privacy and ethical restrictions, but may be available from the corresponding author upon reasonable request and subject to institutional approval.
